# Modulation of NMDA channel gating by Ca^2+^ and Cd^2+^ binding to the external pore mouth

**DOI:** 10.1038/srep37029

**Published:** 2016-11-16

**Authors:** Ya-Chi Tu, Ya-Chin Yang, Chung-Chin Kuo

**Affiliations:** 1 Department of Physiology, National Taiwan University College of Medicine, Taipei, Taiwan; 2 Department of Biomedical Sciences,College of Medicine, Chang Gung University, Tao-Yuan, Taiwan; 3 Graduate Institute of Biomedical Sciences, College of Medicine, Chang Gung University, Tao-Yuan, Taiwan; 4 Neuroscience Research Center, Chang Gung Memorial Hospital, Linkou Medical Center, Tao-Yuan, Taiwan; 5 Department of Neurology, National Taiwan University Hospital, Taipei, Taiwan

## Abstract

NMDA receptor channels are characterized by high Ca^2+^ permeability. It remains unclear whether extracellular Ca^2+^ could directly modulate channel gating and control Ca^2+^ influxes. We demonstrate a pore-blocking site external to the activation gate for extracellular Ca^2+^ and Cd^2+^, which has the same charge and radius as Ca^2+^ but is impermeable to the channel. The apparent affinity of Cd^2+^ or Ca^2+^ is higher toward the activated (a steady-state mixture of the open and desensitized, probably chiefly the latter) than the closed states. The blocking effect of Cd^2+^ is well correlated with the number of charges in the DRPEER motif at the external pore mouth, with coupling coefficients close to 1 in double mutant cycle analyses. The effect of Ca^2+^ and especially Cd^2+^ could be allosterically affected by T647A mutation located just inside the activation gate. A prominent “hook” also develops after wash-off of Cd^2+^ or Ca^2+^, suggesting faster unbinding rates of Cd^2+^ and Ca^2+^ with the mutation. We conclude that extracellular Ca^2+^ or Cd^2+^ directly binds to the DRPEER motif to modify NMDA channel activation (opening as well as desensitization), which seems to involve essential regional conformational changes centered at the bundle crossing point A652 (GluN1)/A651(GluN2).

Ionotropic glutamate receptors mediate the majority of fast excitatory synaptic transmission in the central nervous system. The NMDA receptor channel, one of the major types of ionotropic glutamate receptors, conveys not only electrical but also chemical signals because of its high permeability to Ca^2+^ 1,2,3,4. Ca^2+^ influxes through NMDA receptors lead to many physiological and pathophysiological events, such as synaptic plasticity, gene expression, and cellular damages associated with epilepsy, ischemia, and neurodegenerative disorders^5^,^6^,^7^,^8^,^9^,^10^,^11^,^12^,^13^,^14^.

The NMDA receptor is a heterotetramer composed of two GluN1 and two GluN2 (or GluN3) subunits. Its activation requires binding of two different ligand molecules, namely glycine and glutamate (to GluN1 and GluN2 receptors, respectively). Each subunit contains 3 transmembrane domains (M1, 3 and 4) and a re-entrant loop (M2). The ligand-binding domain consists of two regions termed S1 and S2, which are the N-terminal part following the LIVBP (leucine/isoleucine/valine binding protein)-like domain and the extracellular linker between M3 and M4, respectively^15^,^16^. The intracellular carboxyl terminal domain (CTD) interacts with many signal-transduction and scaffolding proteins^17^. The internal part of the channel pore is formed by the M2 loop whose tip is responsible for ion selectivity^18^, whereas the external vestibule of the pore probably consists of residues in the pre-M1 domain, the C-terminal part of M3 and the N-terminal part of M4^19^,^20^,^21^. There are at least three major gating conformations of the NMDA receptor. Upon binding of gating ligands, the closed (or resting) state would be turned into the open state by opening of an activation gate located presumably at the external pore mouth^22^,^23^,^24^,^25^. With continuous binding of the ligands, however, the channel would tend to be desensitized and become non-conducting to ions again in a steady-state consideration. The location of the desensitization gate is unclear, but one type of desensitization, namely Ca^2+^-dependent desensitization, has been related to conformational changes in the internal vestibule of the channel (“the c-terminal site”)^10^,^26^,^27^,^28^,^29^,^30^,^31^,^32^,^33^. Quite a few molecules such as calcineurin, CaMKIIα, and PSD-95 were reported to be closely associated with the c-terminal site as well as Ca^2+^-dependent desensitization^34^,^35^,^36^.

In addition to the c-terminal site, Ca^2+^ binding may also occur in the external vestibule of the pore such as the DRPEER motif, a highly charged motif located at the extracellular end of M3 in the GluN1 subunit^19^,^20^,^37^ and very close to (~5 residues away from) the activation gate^21^,^23^. In the brain, local concentration of extracellular Ca^2+^ may change profoundly (e.g. from ~2 to ~0.1 mM) and rapidly (e.g. within milliseconds) during normal neuronal and synaptic activities^38^,^39^,^40^. There could be even more robust changes during pathophysiological conditions characterized by extreme neural activities such as epileptic seizures^41^. These fluctuations in the extracellular Ca^2+^ level are at least partly ascribable to the Ca^2+^ influx through the NMDA receptor^42^. In this regard, an extracellular Ca^2+^ binding site, if the occupancy of which could lead to significant gating changes, could then serve to “sense” the extracellular level of Ca^2+^ and act as an intramolecular feedback mechanism to regulate Ca^2+^ influxes and related physiological consequences. Consistent with this view, a recent work combining single-channel recordings and model simulations suggested a lengthened closed time by extracellularly applied Ca^2+^ 43. However, the model does not intend to differentiate the effects of Ca^2+^ binding to an extracellular site from a site in the conduction pathway (pore). Moreover, some of the basic assumptions, such as lack of Ca^2+^ binding to the fully deactivated states of the channel, were left unchecked. In this study, we took advantage of Cd^2+^, a divalent cation with very similar radius and charge density to Ca^2+^ yet impermeable to the NMDA receptor, and demonstrated that external Cd^2+^ as well as Ca^2+^ may bind to the DRPEER motif in both closed and activated conformations with differential affinity, and thus have not only pore-blocking but also gating modification effect. NMDA receptor channel activation (opening as well as desensitization) therefore seems to involve critical conformational changes in the vicinity of the bundle crossing point in the external pore mouth.

## Results

### Reduction in macroscopic currents by external binding of Ca^2+^ and Cd^2+^ to both activated (open as well as desensitized) and closed NMDA channels

**Figure 1:**
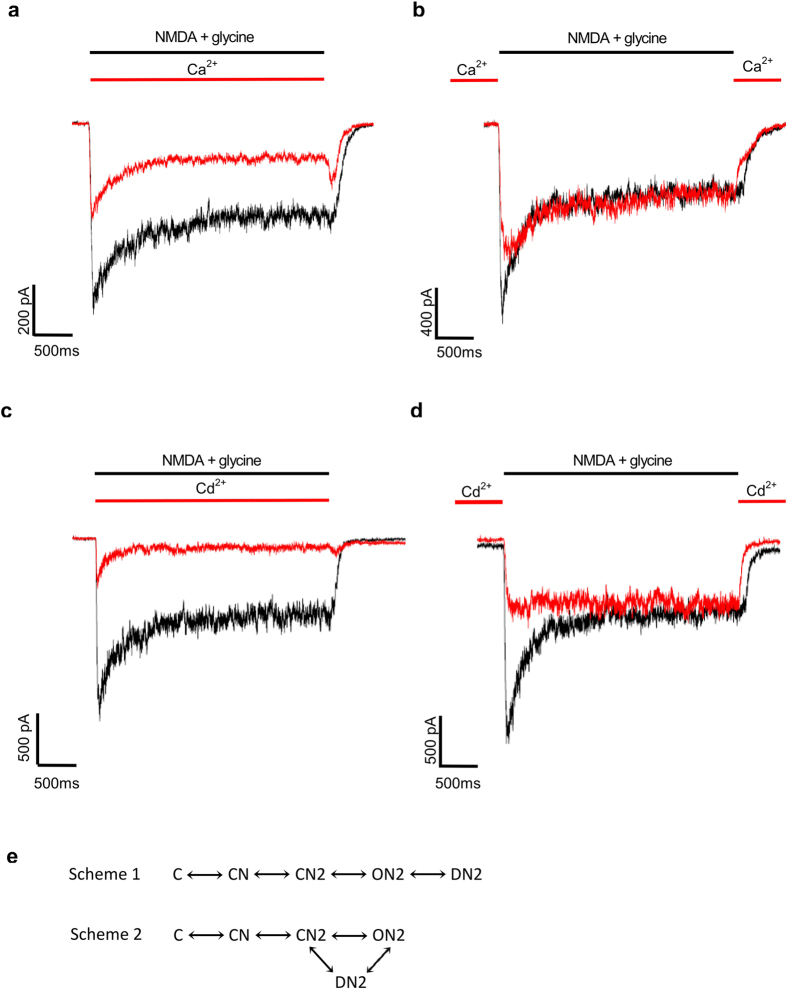
Inhibition of the NMDA receptor currents by extracellular Cd^2+^ and Ca^2+^ presented to different gating states of the channel. (**a**) and (**c**) NMDA receptor currents are elicited by a 3-s pulse of 300 μΜ NMDA plus 30 μΜ glycine (the “NMDA pulse”) every 15 s in the absence (black) or presence (red) of 2 mM extracellular Ca^2+^ (**a**) or 30 μΜ extracellular Cd^2+^ (**c**) during the pulse. (**b**) and (**d**) NMDA receptor currents are elicited by essentially the same protocols in parts a and c, but 2 mM extracellular Ca^2+^ (**b**) or 30 μΜ extracellular Cd^2+^ (**d**) is present between rather than during the NMDA pulses. (**e**) NMDA receptor gating schemes for data analysis (see Methods). N denotes NMDA (assuming the presence of saturating concentrations of glycine). C, CN, and CN2 denote the closed state of the channel, bound with zero, one, and two NMDA molecules, respectively, and ON2 and DN2 are the open and desensitized state of the channel with two bound NMDA molecules, respectively.

Figure 1 shows macroscopic NMDA receptor (NMDAR) currents elicited by brief application of both NMDA and glycine (a NMDA pulse) to an oocyte patch. The presence of 2 mM extracellular Ca^2+^ during the NMDA pulse decreases the NMDAR current (Fig. 1a). The phenomenon by itself could be ascribable to a slower movement of Ca^2+^ (than that of monovalent cations) in the conduction pathway and thus “permeation block” of the NMDA channel pore^4^,^44^. However, application of the same concentration of extracellular Ca^2+^ before, but not during, the NMDA pulse also results in an evident decrease of the NMDAR current peak (Fig. 1b). The action of externally applied Ca^2+^ before the NMDA pulse most likely should be on the closed channel. The action of the externally applied Ca^2+^ during the NMDA pulse, however, could be on either open or desensitized states of the channel. We would simplistically refer to the mixed states as “activated channels” to represent the steady-state mixture of the open and desensitized in the continuous presence of the activating ligands to the channel. Qualitatively very similar effects are obtained by extracellular application of the impermeable ion Cd^2+^ (Fig. 1c and d). There is, however, an interesting difference that there is a much more prominent “hook” current upon simultaneous wash-off of both the blocker and the activating ligands in Fig. 1a than in Fig. 1c. This could implicate a slower unbinding rate of Cd^2+^ than Ca^2+^ (see below). In any case, these findings suggest that Ca^2+^ and Cd^2+^ bind to an extracellular site of the closed NMDAR. The binding then results in interference of ion permeation and/or channel gating to decrease the currents. Cd^2+^ seems to have higher affinity than Ca^2+^ for binding to this external site, because 30 μM Cd^2+^ shows stronger effects than 2 mM Ca^2+^ in the extracellular milieu.

### Cd^2+^ occludes the closed NMDA channel pore from the external site

**Figure 2:**
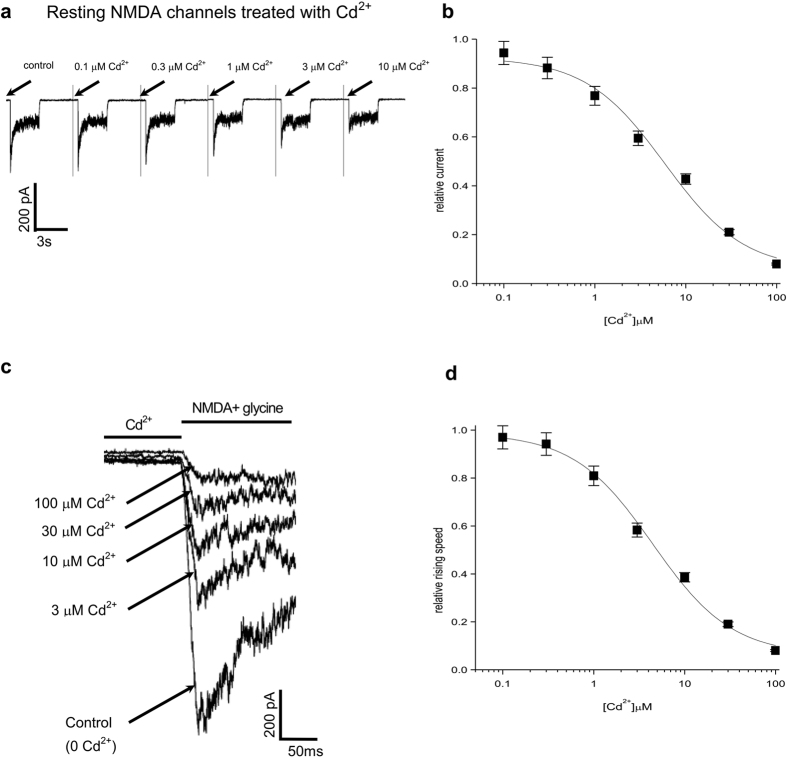
Dose-dependent inhibition of the NMDA receptor currents by Cd^2+^ binding to the closed state of the channel. (**a**) NMDA currents are elicited by essentially the same protocols as in Fig. 1d but with different concentrations of Cd^2+^ applied in the interpulse phase. Cd^2+^ dose-dependently inhibits elicited peak currents, but the late phase of the currents is always essentially unaffected. (**b**) The amplitude of the peak current in Cd^2+^ is normalized to that in control to obtain the relative current, which is plotted against Cd^2+^ concentration (n = 7). The data points are fitted with a Hill equation (equation (5) in Methods) with Kd and n of ~6 μM and 1, respectively. (**c**) The existence of Cd^2+^ in the interpulse phase also decreases the initial activation speed of the macroscopic current in a Cd^2+^ concentration-dependent manner. (**d**) The apparent initial speed of activation in different concentration of Cd^2+^ in part c is normalized to that in control to give the relative activation speed, which is plotted against Cd^2+^ concentration (n = 4). The data points are fitted with a Hill equation (equation (5) in Methods) with Kd and n of ~4.5 μM and 1, respectively.

Because of the similarity of Ca^2+^ and Cd^2+^ effects and the impermeability of Cd^2+^ that restricts its action to the extracellular side of the channel, we then used Cd^2+^ as a probe to characterize the possible extracellular Ca^2+^/Cd^2+^ binding site in the closed NMDAR. Extracellular Cd^2+^ binds to the resting NMDA channel and concentration-dependently inhibits currents with an apparent dissociation constant of 6.0 μM (Fig. 2a and b). As a different approach, the plot of the initial rising speed of the macroscopic current (the apparent activation speed) against Cd^2+^ concentration also gives a similar apparent dissociation constant (i.e. 4.5 μM, Fig. 2c and d). These data are consistent with the idea that bound Cd^2+^ already blocks the pore in the closed channel. Under such circumstances, only the channel not bound with a Cd^2+^ would give rise to currents (ionic fluxes) upon activation (Note the roughly unchanged time to peak of the macroscopic currents by different concentrations of Cd^2+^ in Fig. 2c). If so, the binding site for Cd^2+^ would be most likely located external to the activation/deactivation gate, which is presumably situated in the external pore mouth of the channel^21^,^23^,^45^.

### External Cd^2+^ binds to the activated channel pore with different affinity from that to the closed pore

**Figure 3:**
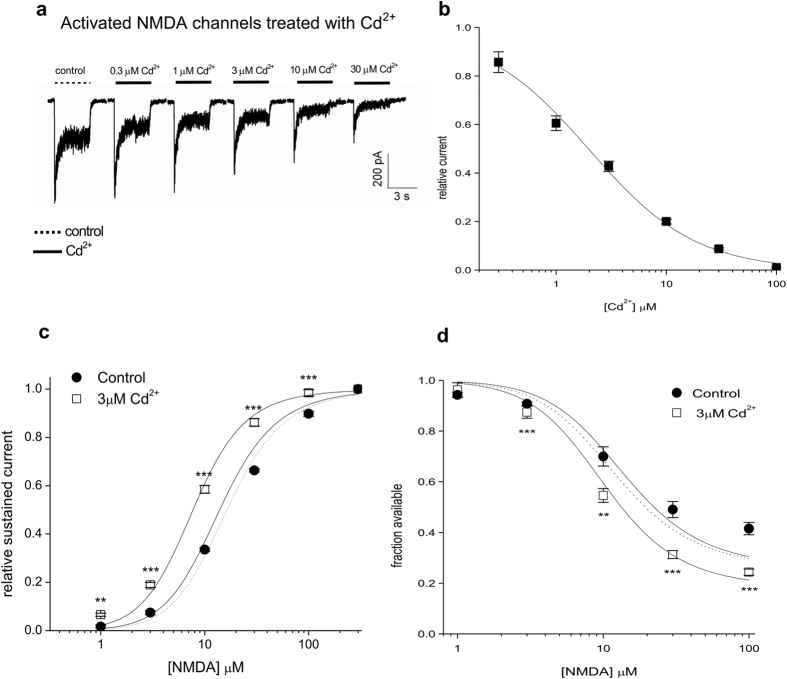
Cd^2+^ binding to the activated NMDA receptor channel with a higher affinity than to the closed channel. (**a**) NMDA currents are elicited by essentially the same protocols as in Fig. 1c but with different concentrations of Cd^2+^ applied during the NMDA pulse. Cd^2+^ shows a dose-dependent inhibition of the peak as well as the sustained NMDA currents during the NMDA pulse. (**b**) The amplitude of the sustained current in Cd^2+^ is normalized to that in control to obtain the relative current, and is plotted against Cd^2+^ concentration (n = 7). The data points are fitted with a Hill equation (equation (5) in Methods) with Kd and n of ~1.6 μM and 1, respectively. The groups of Kd values obtained from fits of each individual data sets in Fig. 2b (7.6 ± 1.2 μΜ), 2d (3.4 ± 0.66 μΜ) and 3b (1.4 ± 0.16 μΜ) are also compared with one-way ANOVA followed by the Bonferroni-Holm test. There is significant difference between Figs 2b and 3b (P = 0.0042), and between Figs 2d and 3b (p = 0.028), but not between Fig. 2b and d (p = 0.065). (**c**) The activation curve is shifted by 3 μM Cd^2+^ (n = 4). P = 0.0087, 1.0 * 10^−5^, 7.8 * 10^−10^, 4.6 * 10^−10^, and 9.9 * 10^−7^ compared with control for NMDA concentrations of 1 to 100 μM, respectively. The curves are fits to the data points in control with equation (1) with a fixed apparent dissociation constant of NMDA (K_N_) of 47 μM (the dotted curve) or 40 μM (the solid curve, see Methods for more details), or to the data points in 3 μM Cd^2+^ with equation (2) with a K_Cd2+,o_ value of 2.5 μM and a r value of 8. (**d**) The desensitization curve is shifted by 3 μM Cd^2+^ (n = 4). P = 0.19, 0.00059, 0.0018, 1.7 * 10^−5^, and 0.00035 compared with control for NMDA concentrations of 1 to 100 μM, respectively. The lines are fits to the data points in control with equation (3) with a fixed Kn value of 35 μM (the dotted curve) or 40 μM (the solid curve, see Methods for more details), or to the data points in 3 μM Cd^2+^ with equation (4) with the same K_Cd2+,o_ and r values given in part c.

We then examined whether the binding affinity of external Cd^2+^ would be changed with channel activation. Cd^2+^ inhibits the NMDA currents in a concentration-dependent manner with an apparent dissociation constant of ~1.6 μM if it is present only in the activated (a steady-state mixture of the open and desensitized) state (Fig. 3a and b). The different affinities of external Cd^2+^ toward the closed and the activated conformation of the NMDA receptor channel indicate possible gating effects of Cd^2+^ in addition to a pore blocking effect. Consistently, Cd^2+^ (3 μM) produces a moderate leftward shift of both the activation curve (Fig. 3c) and the desensitization curve (Fig. 3d) of the NMDA channel. The activation data and desensitization data could be well described by equations (1) to (4) and K_Cd_^2+^_,o_ and K_Cd_^2+^_,d_ (the dissociation constant for Cd^2+^ binding to the open and desensitized NMDA channels) values of 2.5 and 1.25 μM, respectively (see Methods). Because there are two free parameters for fitting, and also because these fitting values could be different with different models or preset values, the foregoing K_Cd_^2+^_,o_ and K_Cd_^2+^_,d_ values should not be rigorously taken as any precise estimates. However, it may be fair to say that the data in Fig. 3c and d could be reasonably described with the overall apparent dissociation constant of Cd^2+^ to the activated (a steady-state mixture of the open and desensitized) channels characterized by a completely different approach in Fig. 3b (1.6 μM). All of the three different measurements in Fig. 3 therefore consistently suggest that Cd^2+^ has modestly higher affinity toward the mixture of activated than the closed states of the NMDA channel.

### External Cd^2+^ probably has a higher affinity to the desensitized than to the open state

**Figure 4:**
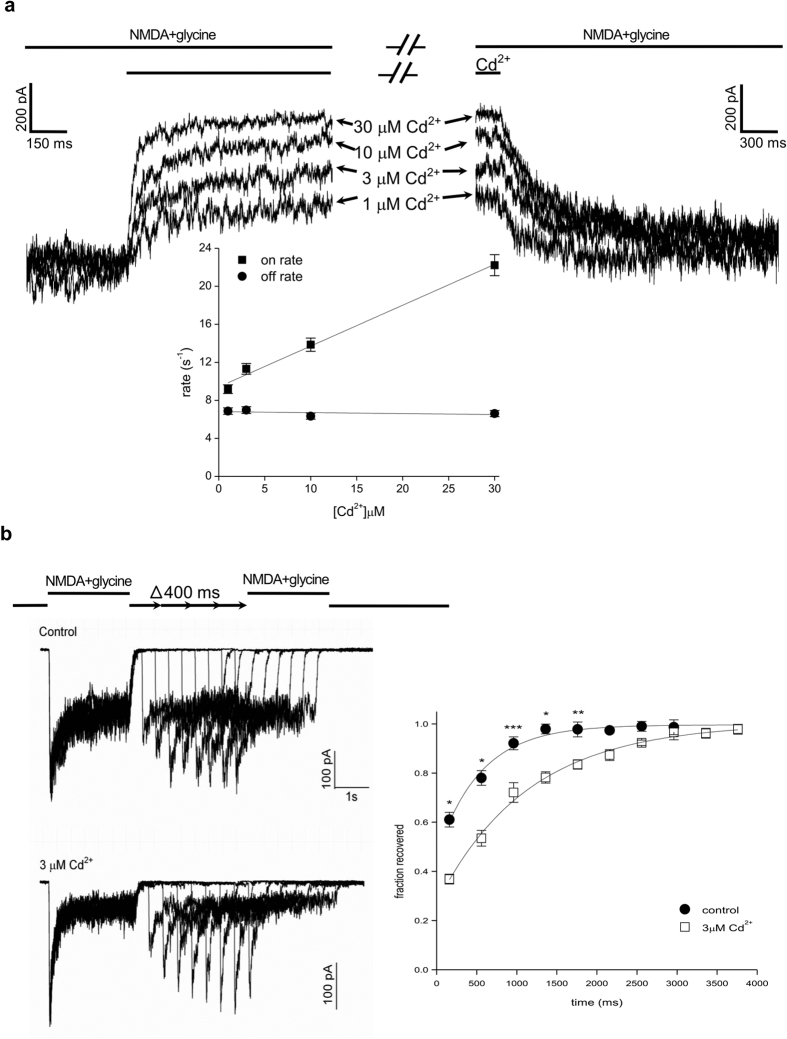
Cd^2+^ binding to the activated NMDA receptor channel via a simple bimolecular process. (**a**) The kinetics of development of and recovery from inhibition of the NMDA currents were studied by fast application and removal of Cd^2+^, respectively, with theta glass tubes. (Inset) Both the decay phase of the current after application of Cd^2+^ and the increment phase of the current after wash-off of Cd^2+^ could be fitted with a monoexponential function to give the binding and unbinding rates (the inverses of the time constants), respectively. The binding and unbinding rates of Cd^2+^ are plotted against Cd^2+^ concentration and fitted with linear regression functions (n = 4). For the binding rates, the slope and intercept are 4.3 × 10^5^ M^−1^s^−1^ and 9.4 s^−1^, respectively. For the unbinding rates, the intercept is 6.8 s^−1^, and the slope is essentially zero (~0.01 M^−1^s^−1^). (**b**) Delayed recovery from desensitization by Cd^2+^. Note the gradually increased NMDA receptor currents in the second NMDA pulse with the lengthening of the recovery period (See Methods for protocols). 3 μM Cd^2+^ is either absent (control, upper panel) or present (lower panel) in the external solution. The difference between the peak current in the second NMDA pulse and the late current in the first NMDA pulse is normalized to the difference between the peak current and the late current in the first NMDA pulse to give the fraction recovered, which is plotted against the duration of the recovery period (right panel, n = 3). The curves are monoexponential fits to the data points with time constants of ~540 and ~1140 ms in control and 3 μM Cd^2+^, respectively. P = 0.027, 0.018, 0.00021, 0.0250, 0.0058, 0.11, 0.16, 0.28, 0.83 and 0.8, respectively, for comparison between the data points of control and 3 μM Cd^2+^ with gradually lengthened recovery time.

Because of the coexistence of the open and desensitized states during the steady-state phase of currents (or in the continuous presence of activating ligands), the affinity of Ca^2+^ or Cd^2+^ specifically toward the open or the desensitized states is difficult to document. We endeavored to explore this intriguing question by the blocking and unblocking rates of Cd^2+^. Although the blocking and unblocking rates are assessed by the kinetics of the decrease and increase of currents through open channels while Cd^2+^ is applied and washed-off, respectively (Fig. 4a), the effect is not necessarily ascribable to the binding and unbinding of Cd^2+^ to and from the open channel. Cd^2+^ binding to the desensitized channels potentially could also alter the occupancy of the channel in the open state. In general, if the transitions between open and desensitized states are faster than the macroscopic binding rates of Cd^2+^, then the apparent binding rates would be a weighted average of the binding rates to the open and desensitized states (weighted by the quasi-steady state occupancy of the desensitized and open states). On the other hand, if the transitions between open and desensitized states are slower than the macroscopic binding rates of Cd^2+^, then the apparent binding rates would be dominated by the binding to the open state. Because in Fig. 4a, the macroscopic on and off rates are generally faster than the macroscopic desensitization and recovery from desensitization (Fig. 4b), respectively, the on and off rates may be predominantly the binding and unbinding rates of Cd^2+^ to and from the open state of the channel. A rough estimate of the dissociation constant of Cd^2+^ to the open state would be ~16 μM (the ratio between the macroscopic off rate and on rates, or 6.8 s^−1^/(4.3 × 10^5^ M^−1^s^−1^), in Fig. 4a, inset). The affinity of Cd^2+^ binding toward the open state therefore may be even lower than toward the closed state. The higher apparent affinity of Cd^2+^ toward the activated channels (a steady-state mixture of the open and desensitized in the continuous presence of activating ligands, Fig. 3) than toward the closed channels (Fig. 2) then would signal a quite higher affinity of Cd^2+^ to the desensitized than to the open states (assuming that the apparent affinity toward the steady-state mixture of the open and desensitized channels would be the weighted average of the individual affinity toward the open and the desensitized channels). Significant binding of 3 μM Cd^2+^ to the desensitized channel could also be qualitatively demonstrated by the slower rate of recovery from desensitization in Cd^2+^ than in control (Fig. 4b). The findings that the macroscopic binding rates are linearly correlated with Cd^2+^ concentration but the unbinding rates are independent on Cd^2+^ concentration would further suggest a simple bi-molecular (one to one) binding process for Cd^2+^ block of the activated NMDA channel pore (Fig. 4a). On the other hand, the linear relationship between the on rate and the concentration of Cd^2+^, the faster on and off rates than the macroscopic desensitization and recovery from desensitization rates^46^, respectively, and the presence of large hook currents upon washing-off of the activating ligands and blocking ions (see below) are also consistent with a concomitant direct pore blocking effect rather than just a gating conformational change (e.g. desensitization) leading to the decreased NMDA currents by external Cd^2+^.

### The DRPEER motif constitutes the binding site for external Cd^2+^

**Figure 5:**
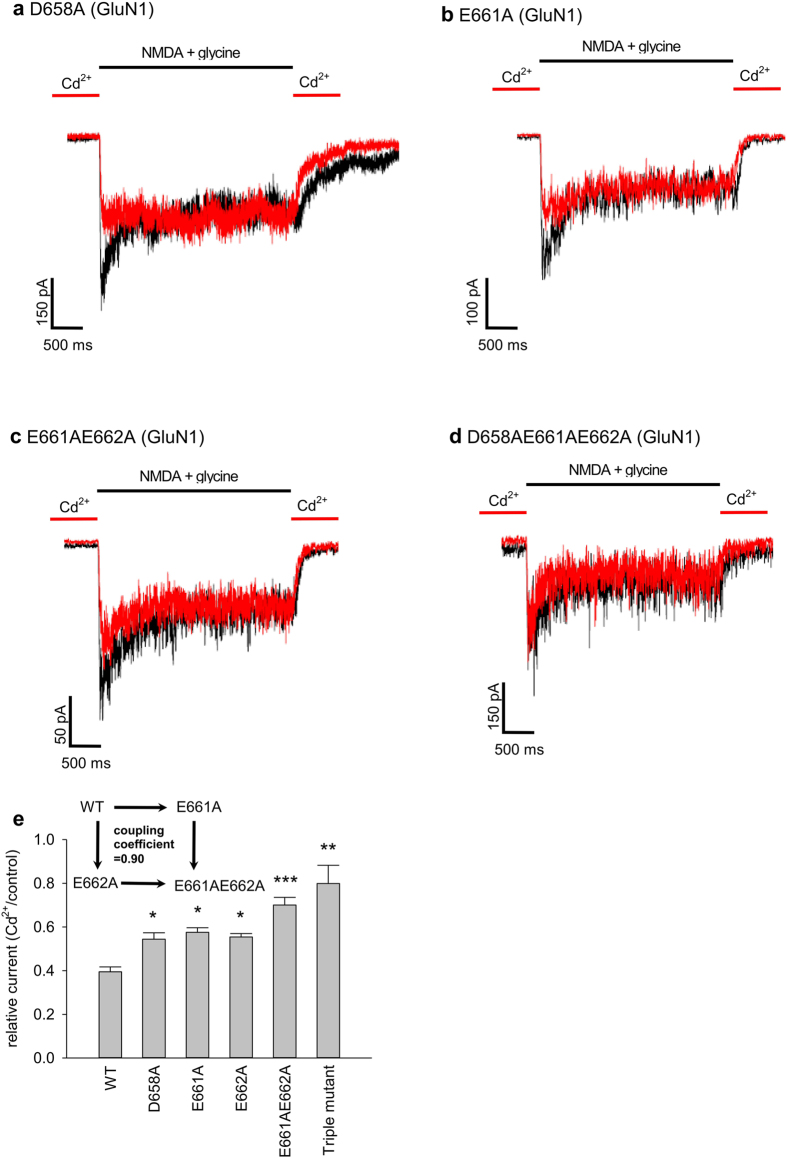
Reduced inhibitory effect for extracellular Cd^2+^ binding to the closed NMDA channel with neutralizing mutations in the DRPEER motif. (**a**) to (**d**) NMDA currents are elicited by the same protocols as that in Fig. 1d. The effect of 30 μM Cd^2+^ is less pronounced in the D658A and in the E661A mutant channels, and even so in the E661AE662A double and D658AE661AE662A triple mutant channels. (**e**) The relative current is defined by the ratio between the peak currents in 30 μM Cd^2+^ and in control (n = 3–13). Note the tendency of reduced Cd^2+^ effect with decreased number of negative charges in the motif. P = 0.034, 0.0013, 0.015, 0.00086, and 0.0093 for D658A, E661A, E662A, E661AE662A double, and D658AE661AE662A triple mutant channels compared with the wild-type (WT) channel, respectively. (Inset) The apparent dissociation constants between Cd^2+^ and the closed wild-type (WT), E661A, E662A, and E661AE662A mutant channels are simplistically derived with the Hill equation (assuming a Hill coefficient of 1, see Fig. 2) and the relative peak currents in 30 μM Cd^2+^, and are 19.6, 40.7, 37.3, and 70.2 μM, respectively. The double mutant cycle analysis shows a coupling coefficient ((Kd_WT_ × Kd_E661AE662A_)/(Kd_E661A_ × Kd_E662A_)) of 0.90 for the two point mutations E661A and E662A in terms of Cd^2+^ binding to the closed NMDA channel.

**Figure 6:**
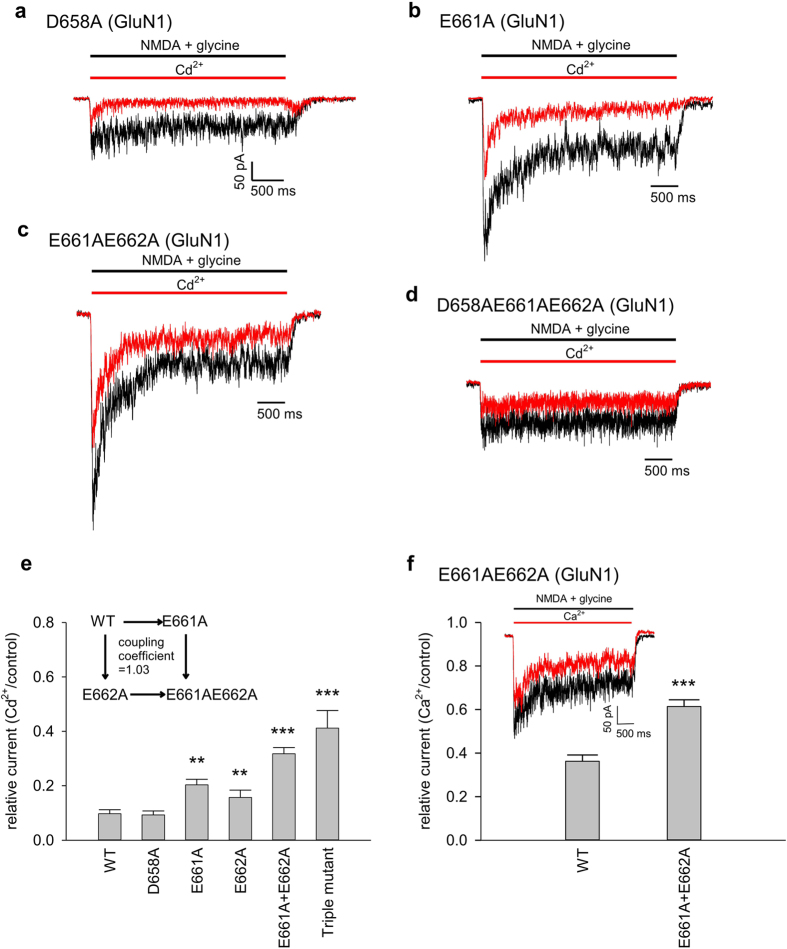
Reduced inhibitory effect for extracellular Cd^2+^ binding to the activated NMDA channel with neutralizing mutations in the DRPEER motif. (**a**) to (**d**) NMDA receptor currents are elicited by the same protocols as those in Fig. 1c. The effect of 30 μM Cd^2+^ is less pronounced in the D658A and in the E661A mutant channels, and even so in the E661AE662A double and D658AE661AE662A triple mutant channels. (**e**) The relative current is defined by the ratio between the sustained currents in 30 μM Cd^2+^ and in control (n = 3–7). Note the tendency of reduced Cd^2+^ effect with decreased number of negative charges in the motif. P = 0.84, 0.0019, 0.0037, 9.1 * 10^−6^, and 0.00031 for D658A, E661A, E662A, E661AE662A double, and D658AE661AE662A triple mutant channels compared with the wild-type (WT) channel, respectively. (Inset) The apparent dissociation constants between Cd^2+^ and the activated wild-type (WT), E661A, E662A, and E661AE662A mutant channels are simplistically derived with the Hill equation (assuming a Hill coefficient of 1, see Fig. 2) and the relative sustained currents in 30 μM Cd^2+^, and are 3.2, 7.7, 5.6, and 13.9 μM, respectively. Double mutant cycle analysis shows a coupling coefficient ((Kd_WT_ × Kd_E661AE662A_)/(Kd_E661A_ × Kd_E662A_)) of 1.03 for the two point mutations E661A and E662A in terms of Cd^2+^ binding to the activated NMDA channel. (**f**) In the E661AE662A double mutant channels, NMDA currents are elicited by the same protocols as those in Fig. 1a (see inset currents). The relative current is defined by the ratio between the sustained currents in 2 mM extracellular Ca^2+^ and in control. The effect of Ca^2+^ is significantly decreased by the E661AE662A double mutation (n = 6; WT: n = 9). ***P = 5.1*10^−5^.

It has been suggested that Ca^2+^ could bind to the external vestibule of the pore such as the DRPEER motif^19^,^20^,^37^. The motif is located external and very close to the activation gate of the NMDA channel, consistent with the prediction based on the biophysical features given above. We therefore replaced the negative charges (i.e. D658, E661, and E662, presumably responsible for binding of Ca^2+^/Cd^2+^) in the motif with alanine, and re-examined the effect of the impermeable Cd^2+^. We found that the effect of Cd^2+^ in either the closed state (Fig. 5) or the activated state (Fig. 6) of the channel is decreased by the neutralizing mutations, and is correlated with the number of charges neutralized. All of the three single charge-neutralization mutations decrease the effect of Cd^2+^, and it is even more so with double and triple mutations (Figs 5e and 6e). The double mutant cycle analysis shows a coupling coefficient close to 1 for the action of Cd^2+^ on either the closed (Fig. 5e) or the activated (Fig. 6e) NMDA channel. These data strongly suggest that the DRPEER motif directly contributes to the binding of external Cd^2+^ in both the closed and the activated channels. Because of the potentially more complicated actions of the permeable Ca^2+^, we did not pursue similar systemic investigations for external Ca^2+^. Perturbation of Ca^2+^ binding is, nonetheless, readily observed with neutralizing mutations in the DRPEER motif (Fig. 6f). In view of the differential binding affinity of Cd^2+^ to the closed and activated states of the NMDA channel (Figs 2, 3, 4), the DRPEER motif very likely undergoes a series of gating conformational changes during channel activation.

### Cd^2+^ and Ca^2+^ produce much larger hook currents upon wash-off in the T647A mutant than in the WT channels

**Figure 7:**
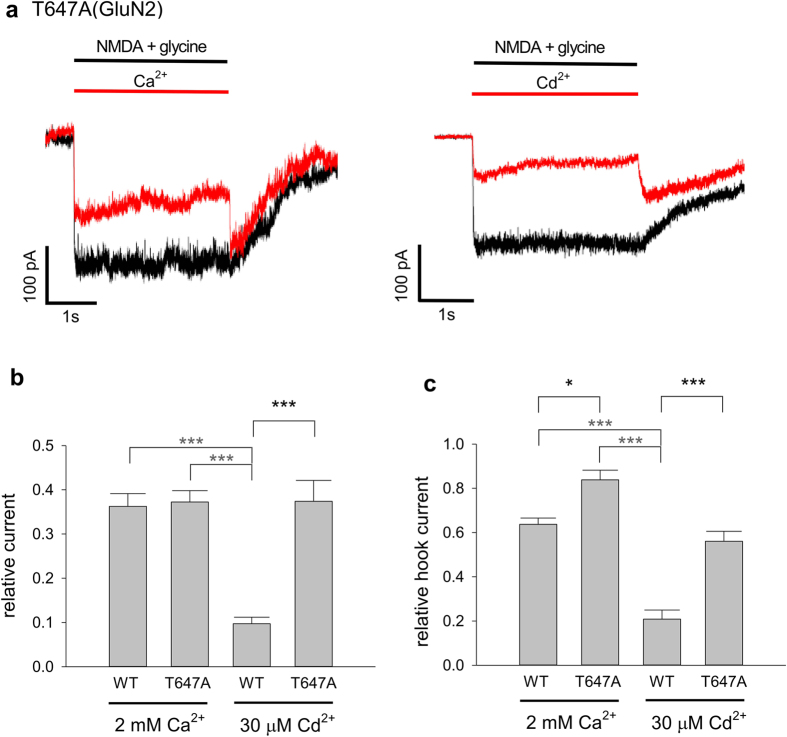
Reduced inhibitory effect of extracellular Ca^2+^ and Cd^2+^ and augmentation of “hook” currents upon wash-off of both the agonists (NMDA and glycine) and the blocking ions in the T647A mutant channel. (**a**) NMDA currents are elicited by the same protocols as those in Fig. 1a and c. The inhibitory effect of Ca^2+^ (left) and Cd^2+^ (right) on the mutant channel currents is reduced (compared to Fig. 1). However, much more prominent “hook” currents immediately after wash-off of both the agonists (NMDA and glycine) and the blocking cations than that in Fig. 1 are noted. (**b**) The relative current is defined by the ratio between the sustained currents in the absence and the presence of the blocking cations. 2 mM Ca^2+^ shows similar effects on the wild-type (WT, n = 9, data from Fig. 6f) and T647A mutant channels (n = 3, P = 0.88). 30 μM Cd^2+^ has a significantly smaller effect on the T647A mutant channel (n = 8) compared to that on the WT channel (n = 6, data from Fig. 6e, P = 0.00037). (**c**) The relative hook current is defined by the ratio between the current peak after wash-off of both the agonists and the blocking cations and the sustained current right before the wash-off. N numbers are the same as those in part b. Both 2 mM Ca^2+^ (P = 0.028) and 30 μM Cd^2+^ (P = 0.00012) produce a significantly larger hook current in the T647A mutant channel than in the WT channel.

We previously reported that felbamate, an anticonvulsant acting as a pore blocker and a gating modifier of the NMDA channel, should bind to V644/T648 (GluN1) and L643/T647 (GluN2B) just inside the activation gate of the channel^24^. We also found that T647A point mutation has a prominent effect on NMDA channel gating. The proportion of constitutively open channels (the open channels in the absence of NMDA and glycine) is significantly increased, whereas channel desensitization is apparently diminished, by the mutation^23^ (also see Fig. 7a compared to Fig. 1). Similar to felbamate, Cd^2+^ also blocks the open channel pore and modestly promotes desensitization (Figs 3 and 4). We therefore studied the effects of extracellular Cd^2+^ in the T647A (GluN2B) mutant channels. Similar to the case of felbamate, the inhibitory effect and the binding affinity of Cd^2+^ is markedly decreased with the T647 mutation (Fig. 7a and b). In addition, a prominent “hook” current develops after wash-off of both the agonists (NMDA and glycine) and Cd^2+^ (Fig. 7a and c). We also repeated the same experiments with extracellular Ca^2+^. Although the blocking effect of Ca^2+^ on the currents is not apparently changed by the mutation, wash-off of Ca^2+^ gives rise to an even larger hook current in the mutant channel (Fig. 7a and c). The markedly different inhibitory effect of Cd^2+^ on the wild-type and T647A mutant channel (Fig. 7b) implicates lowered binding affinity of Cd^2+^ (presumably to the DRPEER motif) by T647A. Consistently, the unbinding of the blocking Ca^2+^ and especially Cd^2+^ may also be faster in the T647A mutant than in the wild-type channel, so that the hook following simultaneous wash-off of both the activating ligands and the blocker is much larger in the mutant than in the wild-type channels. It is interesting that a mutation (at T647) inside the activation gate A651/A652 actually alters the conformation (at the DREPPR motif) outside the gate. NMDA receptor channel activation most likely involves a regional conformational changes centered at rather than limited to the bundle crossing point or the so-called activation gate at A651/A652 (see Discussion).

## Discussion

### Ca^2+^ and Cd^2+^ binding to the DRPEER motif to block ion conduction through the NMDA channel pore

**Figure 8:**
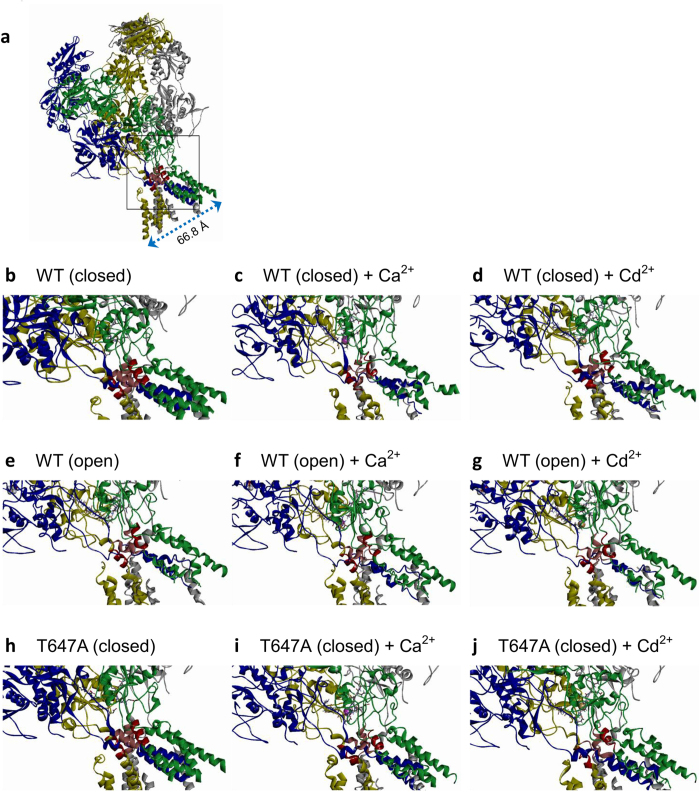
Molecular modeling of the NMDA channel, with emphasis on the region of the SYTANLAFF and DRPEER motifs. The four different subunits of the NMDA channel are illustrated in four different colors, respectively (GluN1: grey and blue; GluN2: yellow and green), except that the SYTANLAFF motifs (the bundle crossing or the presumable activation “gate” regions) in GluN1 and GluN2 are colored in pink and red, respectively. The side chains of the DRPEER motifs (in GluN1 subunits) are shown in ball-and-stick sketches. The Ca^2+^ and Cd^2+^ ions are colored in purple and dark yellow, respectively. (**a**) The whole closed wild-type (WT) NMDA channel. The boxed area is enlarged for the following figures, which are also visualized with the same orientation. One may use the pitch of the α-helices (5.4 Å) as a scale, but should be aware that the figures are just two-dimensional presentations of three-dimensional structures. (**b**–**d**) The closed WT NMDA channel in control (**b**), or with Ca^2+^ (**c**) or Cd^2+^ (**d**) binding to the DRPEER motifs. Note the shapely α-helical structures in the SYTANLAFF motifs and vicinity in the control condition, and the evident conformational changes (e.g. disruption of helices into loops) induced by Ca^2+^ or Cd^2+^ binding. (**e**–**g**) The open/desensitized WT NMDA channel (i.e. the most stable conformation of the channel in the presence of 2 glutamates and 2 glycines, thus most likely a “mixed” conformation of both open and desensitized states) in control (**e**), or with Ca^2+^ (**f**) or Cd^2+^ (**g**) binding to the DRPEER motifs. Note the similarities in the conformational changes in the SYTANLAFF motifs and vicinity in part e to those in parts c and d. Also, Ca^2+^ or Cd^2+^ binding induces much less conformational changes in the open/desensitized than in the closed channels. (**h**–**j**) The closed T647A mutant channel in control (**h**), or with Ca^2+^ (**i**) or Cd^2+^ (**j**) binding to the DRPEER motifs. Note the differences in the conformational changes induced by Ca^2+^ and Cd^2+^ binding (e.g. different patterns of disruption of the helices into loops) between the mutant and the WT channel.

We have shown that Ca^2+^ and Cd^**2+**^ bind to both closed and activated NMDA channels to affect both ion permeation and channel gating. The binding site is therefore located external to the activation gate (presumably at A652 (GluN1)/A651(GluN2))^21^,^23^ and most likely in the DRPEER motif. The DRPEER motif (D658-R663) is a short peptide segment just ~5 amino acids external to the highly conserved SYTANLAFF in GluN1 (but not GluN2). It has been demonstrated that DRPEER motif may confer the high Ca^2+^ flux rate in the NMDA channel, as the relative Ca^2+^ permeability is markedly reduced with neutralization of all of the three negatively charged residues in the motif^37^. In the meanwhile, the effect of 1 mM Ca^2+^ in decreasing the overall current flow through the NMDA channel is also reduced in the triple mutant channel (from 56 to 45 pS in the mutant vs. from 73 to 41 pS in the wild-type channel). Consistently, we found that extracellular Ca^2+^ or Cd^2+^ has a blocking effect on ion permeation through the pore (Figs 1, 2, 3, 4), and all three negatively charged residues in the motif directly contribute to this binding (Figs 5 and 6). Although the DRPEER motif is already located beyond the narrowest bundle-crossing point and at the very external part of the NMDA channel pore, this region seems to remain functionally “narrow” enough to prohibit independent movement of Cd^2+^ or Ca^2+^ and the other permeating ions (see the simulation results in Fig. 8).

### The functional design of the DRPEER motif in GluN1 for NMDA channel gating

In addition to block of ion permeation, we found a novel effect on channel gating with a one-to-one binding process for Cd^2+^ or Ca^2+^ binding to the DRPEER motif. The activated states (probably chiefly the desensitized state) of the NMDA channel are favored by binding of these divalent cations (Fig. 4, and also see the molecular modeling in Fig. 8, where Ca^2+^ or Cd^2+^ binding induces less conformational changes in the presence than in the absence of the activating ligands, consistent with higher affinity of the cations to the DRPEER motifs in the activated or a steady-state mixture of the open and desensitized channels). It would be worthy to note that the DRPEER motif is present only in GluN1 but not in GluN2 and that GluN1 and GluN2 play differential role in NMDA channel gating. We have previously reported that GluN1 assumes a more global control in NMDA channel and move first upon NMDA channel activation. GluN2, which is directly responsible for the channel gate, is then allowed to move to open the channel pore^45^ (also see below). DRPEER contains 5 charged amino acids in a 6-residue stretch, and is located right at the junction between the helix containing the SYTANLAFF motif (which comprises the center of the activation gate and felbamate binding site)^23^,^24^ and a subsequent loop structure^21^. With a secondary structure of a loop or a disintegrating α-helix (at least partly ascribable to the kink at the proline residue), the three negative residues in the DRPEER motif could all face the permeating or blocking Ca^2+^ or Cd^2+^ ion in the pore (Fig. 8). The two arginines, on the other hand, most likely face away from the pore and could be responsible for the interaction with the other peptide chains of the channel. The arginines thus may play two correlative functional roles, maintenance of local conformation and transduction of conformational changes to and from the microenvironment of the motif. The design is reminiscent of the single positively charged residue (lysine) in the DEKA selectivity filter in Na^+^ channels^47^,^48^. The arginines and the proline, on the other hand, may make a relatively fixed orientation of the side chains of adjacent negatively charged amino acids, and thus more “strain” is produced when the negatively charged side chains are abducted by Ca^2+^ or Cd^2+^. The binding affinity of Ca^2+^ or Cd^2+^ thus would be much lower than that toward the EEEE selectivity filter of the Ca^2+^ channel^49^,^50^. Given a one-to-one binding process, this lower affinity may straightforwardly assure a faster unbinding rate of the bound cation (especially Ca^2+^ in the physiological conditions), making a gating modification effect swiftly adaptable to the ions in the microenvironment.

### The scope of Ca^2+^-dependent desensitization of the NMDA channel

The Ca^2+^-dependent modulation of NMDA channel gating is mostly assumed to happen in the intracellular domain, which may interact with Ca^2+^-dependent proteins or enzymes^51^,^52^. In this regard, it is interesting that extracellular Ca^2+^ could reduce single NMDA channel conductance, in addition to a decrease in opening probability, which is chiefly ascribable to the increase in prolonged closed events^43^. If these prolonged closed events, which are in general a few seconds in length, could be viewed as the desensitized state, then these single channel findings would be consistent with the higher affinity of extracellular Cd^2+^ toward the desensitized than the open state demonstrated in this study (Fig. 4). Because Ca^2+^ is a permeant ion of the channel, it is hard to limit the localization of “extracellular Ca^2+^ action” to the extracellular part of the channel protein. The action of extracellular Ca^2+^ on the closed NMDA channel is the first piece of evidence suggesting modulation of NMDA channel gating by Ca^2+^ on the extracellular side (Fig. 1). The action of extracellular Cd^2+^, a cation impermeable to the NMDA channel but with the same size and charge as Ca^2+^ (and usually much stronger affinity to a Ca^2+^ binding site), would thus greatly contributes to the exploration whether modification of channel gating could happen with Ca^2+^ binding to the extracellular part of the channel. The findings that Ca^2+^ or Cd^2+^ binding to the DRPEER motif would favor channel activation (probably more desensitization than opening) may broaden the scope of Ca^2+^-dependent desensitization of the NMDA channel to involve essential conformational changes in the external pore mouth of the NMDA channel, where the activation gate and the glycine-independent desensitization process are very likely also co-localized^23^,^24^,^53^. Together with the findings that the prolonged closed events could always lead into an open event^43^, and that closed NMDA channels could also desensitize^54^, it seems that at least a desensitization state is reciprocally interconnected with both the closed and the open state. It may be desirable to explore the conformational changes near the bundle crossing region at the external pore mouth in more detail for further characterization of the molecular nature of NMDA channel opening and desensitization.

### Physiological implications

NMDA channels convey two messages into the neuron. The electrical signal is carried by cationic influxes chiefly ascribable to monovalent cations. The chemical signal, on the other hand, is carried by Ca^2+^ influxes. We have demonstrated that Cd^2+^ and Ca^2+^ binding to the DRPEER motif could decrease NMDA currents (or more precisely, NMDA currents carried by monovalent cations). On the other hand, this binding could increase the apparent affinity of NMDA to the channel and thus leftward shift of the activation and desensitization curves (Fig. 3). The wide range of extracellular Ca^2+^ concentrations may thus be viewed as a key control signal itself for Ca^2+^ influxes into the cell, which would also be finely tuned by the ambient NMDA concentrations. Moreover, Watanabe *et al*.^37^ has also shown that neutralization of the negative charges in DRPEER would significantly decrease the permeability ratio between Ca^2+^ and Cs^+^, implying Ca^2+^ binding to this motif may contribute to the high flux rates of Ca^2+^ through the channel. It is conceivable that there could be an “electrochemical dissociation effect” of Ca^2+^ binding to the DRPEER motif. The reduced electrical signal may require more convergent glutamatergic synapses to be activated before the same fast electrophysiological postsynaptic effect is achieved. In the meanwhile there would be more chemical signal or Ca^2+^ influxes into the cell. This augmented chemical signal for the same electrical signal may thereafter give rise to physiological or even pathophysiological consequences related to the increase of intracellular Ca^2+^ mediated by the NMDA channel.

## Methods

### Molecular biology

The rat GluN1a variant and GluN2B cDNA clones were used in this study. Point mutations were made by site-directed mutagenesis using the QuickChange Site-Directed Mutagenesis kit (Stratagene, La Jolla, CA). Mutations were verified by DNA sequencing to ensure the lack of any inadvertent mutations. The capped cRNA transcripts were then synthesized using T7 and T3 mMESSAGE mMACHINE transcription kits (Ambion, Austin, TX), and were stored at −80 °C.

### Preparation of oocytes

This study was carried out in accordance with the ethical information guidelines of the National Taiwan University College of Medicine. The protocols for the care and use of animals were approved by the National Taiwan University College of Medicine and College of Public Health Institutional Animal Care and Use Committee (IACUC). All efforts were made to minimize animal suffering. Adult female *Xenopus laevis* were anesthetized, and ovarian lobes containing stage V or VI oocytes were removed. Oocytes were dispersed after manual disruption of the egg sac, and were digested for 1 hr with collagenase type I (2 mg/ml) to remove the follicular layer. A mixture of GluN1a variant and GluN2B cRNAs with a ratio of 1:5 (i.e., 0.1–4 ng of GluN1a variant and 0.5–20 ng of GluN2B) (Kashiwagi *et al*. 2002) was injected into oocytes. Oocytes were maintained in the culture medium (96 mM NaCl, 2 mM KCl, 1.8 mM MgCl_2_, 1.8 mM CaCl_2_, 5 mM HEPES, and 50 ug/ml gentamycin, pH 7.6) at 18 °C for 2–3 days before electrophysiological recordings.

### Outside-out patch recordings

Oocytes were placed in a recording chamber containing Mg^2+^-free bath solution (150 mM NaMeSO_3_, 5 mM NaCl, and 10 mM HEPES; pH 7.4). Outside-out patch recordings were obtained using fire-polished pipettes pulled from borosilicate capillaries (outer diameter, 1.55–1.60 mm; Hilgenberg, Malsfeld, Germany). Recording pipettes (0.1~0.2 MΩ) were filled with the pipette solution containing 150 mM NaMeSO_3_, 5 mM NaCl, 5 mM EGTA, and 10 mM HEPES (pH 7.4). A seal was formed, and the outside-out patch configuration was obtained in Tyrode’s solution (150 mM NaCl, 4 mM KCl, 2 mM MgCl_2_, 2 mM CaCl_2_, and 10 mM HEPES; pH 7.4). The patch was then lifted from the bottom of the chamber and moved in front of a set of “theta glass” tubes (2.0 mm outer diameter pulled to an opening of ~300 μm in width; Warner Instrument, Hamden, CT) emitting external recording solutions. The standard external solution was Mg^2+^-free Ca^2+^-free Tyrode’s solution (pH 7.4), but 2 mM Ca^2+^ or 0.1–100 μM Cd^2+^ may be added to the external recording solution as specified in each different experimental condition. The glass-tube holder was connected to a stepper motor (SF-77B perfusion system, Warner Instrument) to achieve fast switch for rapid solution change. Drugs and salts were purchased from Sigma (St. Louis, MO). NMDA, glycine, and Cd^2+^ were dissolved in water to make 100, 100, and 10 mM stock solutions, respectively. The stock solutions were then diluted into the external recording solution to make 1 to 100 μM Cd^2+^, 1 to 300 μM NMDA, and 30 μM glycine. The NMDA currents were recorded at a membrane potential of −70 mV and at room temperature (~25 °C) with an Axoclamp 200A amplifier, filtered at 1 kHz with a four-pole Bessel filter, digitized at 500 μs intervals, and stored using a Digidata-1322A analog/digital interface along with the pCLAMP software (all from MDS Analytical Technologies, Sunnyvale, CA). To plot the activation and the desensitization curves, a patch was moved from the NMDA- and glycine-free external solution to the external solution containing 0 to 100 μM NMDA and 30 μM glycine for 3 s (the desensitization prepulse), and then moved to another external solution containing 300 μM NMDA and 30 μM glycine for 3 s (the test pulse) before being moved back to the NMDA-free external solution again. The amplitude of the sustained currents in the desensitization prepulse in each different concentration of NMDA is normalized to the maximal current and plotted against the NMDA concentration to obtain the activation curve. For the plot of the desensitization curve, the amplitude of the peak currents in the test pulse is normalized to the maximal current (with 0 μM NMDA in the desensitization pulse) to get the relative available current, which is then plotted against the NMDA concentration in the desensitization pulse. For measurement of the kinetics of recovery from desensitization, a patch was moved from the NMDA- and glycine-free Tyrode’s solution to the external solution containing 300 μM NMDA and 30 μM glycine for 2 s (the first NMDA pulse), and then moved to the NMDA- and glycine-free Tyrode’s solution for different durations (the “recovery period”) before being moved back to the external solution containing 300 μM NMDA plus 30 μM glycine (the second NMDA pulse) to elicit NMDA receptor currents.

### Data analysis

As a first approximation, the two NMDA sites are assumed to have the same binding affinity, and the binding of one ligand will not affect the binding of the other. We may then have two simplified gating schemes (Fig. 1e)^55^. Although the NMDA channel may be desensitized without opening after binding of two NMDA molecules (i.e. Scheme 2 in Fig. 1e)^54^,^56^, there is no substantial difference between the two schemes in steady-state considerations. We therefore chose to use a linear basic C-O-D model (Scheme 1 in Fig. 1e) for further analysis for simplicity.

Let p be the relative chance that the channel is open vs. staying closed when both sites are occupied by agonist NMDA (i.e. ON2/CN2), and m is the ratio between the steady-state occupancy of the desensitized and the open states (i.e. DN2/ON2). The relative steady-state current (I) in the presence of saturating concentrations of glycine and the absence of Cd^2+^ would then be a function of the NMDA concentration, and can be described in the following form^46^,^55^:


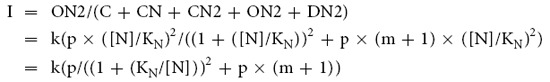


where [N] is the concentration of NMDA, and K_N_ is the dissociation constant of NMDA.

When [N] is very large, then I approaches its maximal value I_max_, and


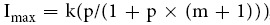
, the relative steady-state current (I/I_max_) therefore would be:


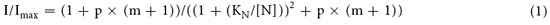


In addition, the relative steady-state current in the presence of saturating concentrations of glycine and a fixed concentration of Cd^2+^ would be:


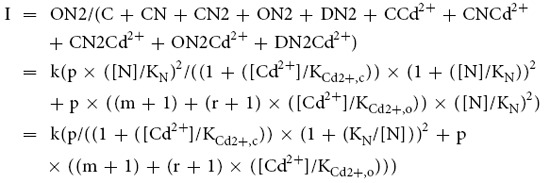



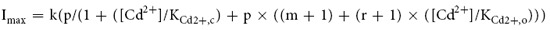



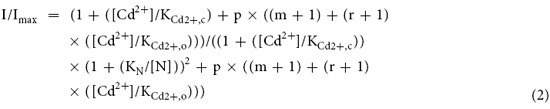


where CCd^2+^, CNCd^2+^, CN2Cd^2+^, ON2Cd^2+^, and DN2Cd^2+^ denote states C, CN, CN2, ON2, and DN2 bound with a Cd^2+^ ion. [Cd^2+^] is the concentration of Cd^2+^, and K_Cd2+,c_ and K_Cd2+,o_ are the dissociation constants between Cd^2+^ and the closed and open NMDA channel, respectively.

On the other hand, the proportion of the available channel in the continuous presence of different concentrations of NMDA (i.e. the desensitization pulse) would be defined by the relative current elicited at the subsequent pulse with saturating concentration of NMDA, and can be described as:


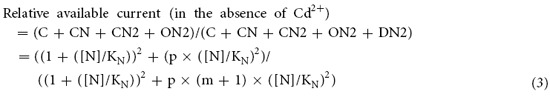



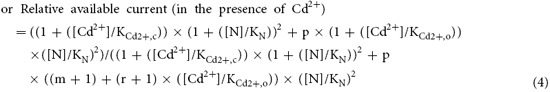


where r = m × (K_Cd2+,o_/K_Cd2+,d_). For the sake of simplicity, the p and m values are set at 2.6 and 4, respectively^46^. We then obtained the K_N_ value from the fits to the activation and desensitization data in the absence of Cd^2+^ in Fig. 3 (47 μM and 35 μM, respectively, a K_N_ value of 40 μM is therefore used for subsequent analysis). Moreover, K_Cd2+,c_ is set at 5 μM based on the fits to the data in Fig. 2 with the Hill equation:


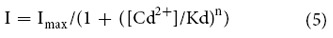


where Kd is the dissociation constant of Cd^2+^ binding to the NMDA channel (e.g. if the channel is in the closed state, then the Kd would be K_Cd2+,c_).

With the foregoing values of p, m, K_N_, and K_Cd2+,c_, we obtained K_Cd2+,o_ and r values (2.5 μM and 8, respectively) from the fits to the activation and desensitization data in the presence of Cd^2+^ in Fig. 3. K_Cd2+,d_ therefore would be equal to K_Cd2+,o_ × m/r, or 1.25 μM. All averaged data are expressed as mean ± SEM. The data in Figs 3c,d, 4b and 6f are compared using two-tailed unpaired Student’s *t* tests, whereas all the other data are compared with one-way ANOVA followed by the Bonferroni-Holm test for multiple comparisons. Exact P values are reported. *denotes p < 0.05, **denotes p < 0.01, and ***denotes p < 0.001 in the figures.

### Homology modeling of the NMDA channel

The NMDA channel homology modeling was built based on the X-ray crystal structural data of the Xenopus NMDA channel reported by Lee *et al*.^21^, the only structural data of the NMDA channel so far. The DRPEER motif in rat GluN1 subunit, however, is RRPEER in the Xenopus GluN1 subunit. We therefore replaced the arginine in the Xenopus sequence with glutamate before the actual modeling process. Molecular dynamics simulation for modeling of the WT (PDB ID: 4TLL) and T647A mutant NMDA channels were performed using Discovery Studio v2.5 programs (Accelrys Inc., San Diego, CA USA) and the chemistry at Harvard Molecular Mechanics (CHARMm) force field. The simulation system provides a “steepest descent” energy minimization with positional restraints. Ca^2+^ or Cd^2+^ was introduced at the highest electrostatic potential in the DRPEER region. A second conjugated gradient energy minimization was then performed until no significant energy change could be detected. Berendsen coupling was applied for the simulation parameters to maintain a constant temperature of 300 °K and a constant pressure of 1 bar. The Van der Waal’s force was modeled with a cutoff value of 10 Å. The Leapfrog Verlet procedure was used to integrate the equation of motion, and the one with the lowest potential energy for equilibration was selected from ~5 candidate models.

## Additional Information

**How to cite this article**: Tu, Y.-C. *et al*. Modulation of NMDA channel gating by Ca^2+^ and Cd^2+^ binding to the external pore mouth. *Sci. Rep.* **6**, 37029; doi: 10.1038/srep37029 (2016).

**Publisher's note**: Springer Nature remains neutral with regard to jurisdictional claims in published maps and institutional affiliations.
